# Mediastinal neoplasms in patients with Graves disease: a possible link between sustained hyperthyroidism and thymic neoplasia?

**DOI:** 10.1186/1756-6614-5-5

**Published:** 2012-07-23

**Authors:** Jonathan D Boyd, Ridas Juskevicius

**Affiliations:** 1 Department of Pathology & Laboratory Medicine, Vidant Medical Center and Brody School of Medicine, East Carolina University, Greenville, NC, USA; 2 Department of Pathology & Laboratory Medicine, Brody School of Medicine, East Carolina University, 600 Moye Blvd, Brody Medical Sciences Building 7S18, Greenville, NC, 27858-4353, USA

**Keywords:** Graves disease, Hyperthyroidism, Thymoma, T Lymphoblastic leukemia/lymphoma, Thymic hyperplasia

## Abstract

**Background:**

Anterior mediastinal masses are a rare but well documented finding in Graves disease. The vast majority of these lesions represents benign thymic hypertrophy and regress after treatment of the hyperthyroidism. A small percentage of these cases however represent neoplastic/malignant diseases which require further treatment.

**Cases:**

12 year old boy with one year history of refractory Graves disease was found to have an anterior mediastinal mass and underwent curative thyroidectomy for sustained hyperthyroidism. Cervical lymphadenopathy was detected during the procedure and biopsy was obtained. A 23 year old woman who presented with a one month history of hyperthyroid symptoms, was diagnosed with Graves disease and also was found to have an anterior mediastinal mass on imaging. Biopsy of the anterior mediastinal mass was obtained and subsequently the patient underwent robotic thymectomy. Histologic examination and immunophenotyping of the cervical lymph node in a 12 year old boy revealed neoplastic proliferation of T lymphoblasts diagnostic of T lymphoblastic leukemia/lymphoma. Examination of the anterior mediastinal mass biopsy in the 23 year old woman revealed type B1 thymoma which was confirmed after examination of the subsequent robotic thymectomy specimen.

**Conclusion:**

This is the first reported case of T cell lymphoblastic lymphoma and the third reported case of thymoma associated with sustained hyperthyroidism due to Graves disease. These cases indicate that an anterior mediastinal mass in a patient with active Graves disease may be due to a neoplastic cause, which may require definitive treatment. Caution should be exercised when dismissing a mediastinal mass as benign thymic hyperplasia in patients with active Graves disease.

## Background

Graves disease (GD) is an autoimmune disease caused by self-reactive plasma cells which produce antibodies to the thyrotropin receptor that stimulate thyroid-stimulating hormone receptors and increase the production of thyroid hormone [1]. The relationship between Graves disease (GD) and mediastinal masses is nearly a century old having been first identified in a patient with thymic hyperplasia secondary to GD described by Halsted in 1914 [2]. With a well known benign cause of anterior mediastinal masses (AMM) in Graves disease, there is a tendency to forego tissue biopsy and re-evaluate at an unspecified time after thyroid ablation/thyroidectomy [2-5]. Here we describe two patients with neoplastic anterior mediastinal masses associated with GD, illustrating that this may not be safe approach to all patients with anterior mediastinal masses and GD.

## Case presentations

### Patients

**Figure 1:**
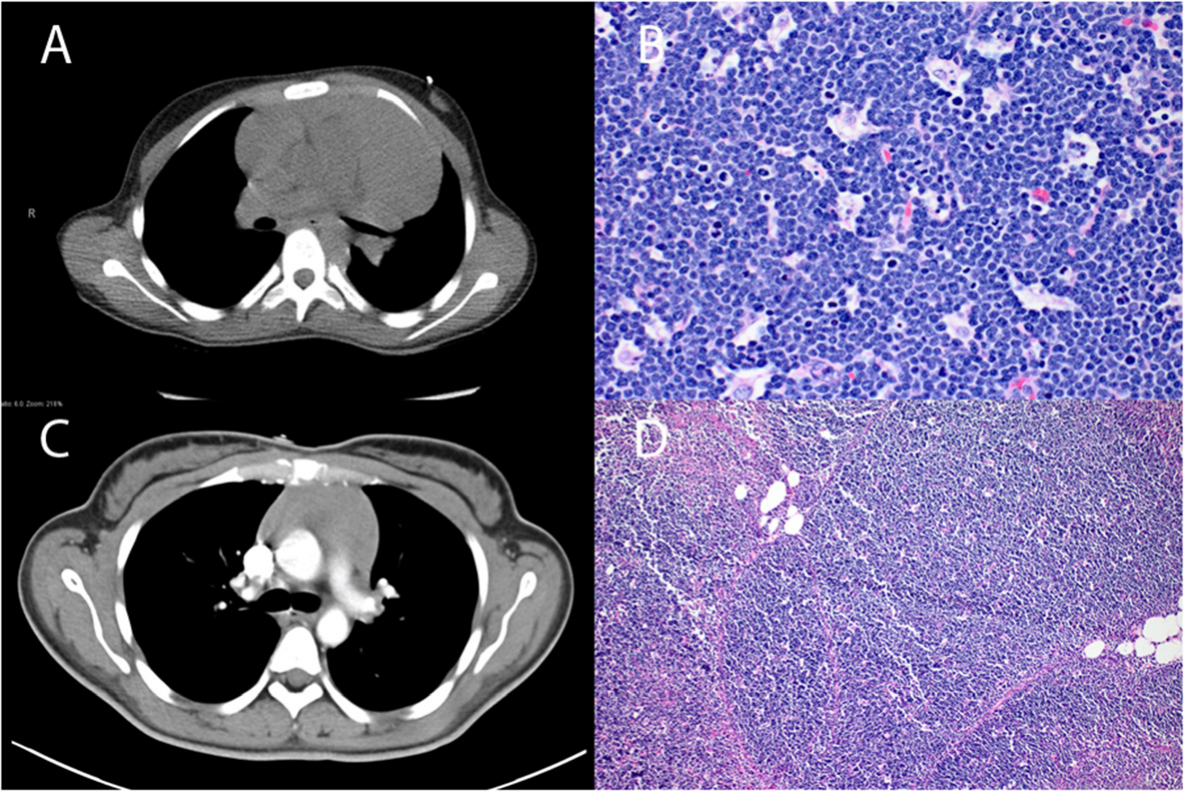
**(A and C) Chest computed tomography images showing anterior mediastinal masses from patients with T-cell LBL/L and Type B1 thymoma.** (**B** and **D**) Representative tissue sections from the same patients (Hematoxylin-Eosin, x200).

A 12 year-old African American male with a one year history of GD refractory to medical management presented with a persistent tachycardia, fever, abdominal pain, vomiting, weakness and muscle aches after discontinuing methimazole in preparation for thyroid radioablation. A 12 × 6 cm mediastinal mass had been identified radiographically which was initially thought to represent thymic hyperplasia (Figure 1A). Bilateral cervical lymphadenopathy was also detected. The decision was made to perform a curative thyroidectomy. During the procedure a cervical lymph node biopsy was performed, which demonstrated architectural effacment by a diffuse infiltrate of immature lymphoid cells (Figure 1B). The immunophenotyping by flow cytometry revealed a predominance of T lymphoblasts (CD1a+, CD2+, CD4+, CD8+, sCD3-, TdT+) consistent with T-lymphoblastic leukemia/lymphoma (T-LBL/L). Bone marrow examination identified involvement by T-LBL. The thyroidectomy specimen contained changes typical of GD. The patient was treated with induction chemotherapy for T-LBL with excellent response showing essentially complete resolution of the mediastinal mass, neck lymph nodes and no bone marrow involvement. Patient has continued to do well post thyroidectomy on levothyroxine.

The second patient is a 23-year old female without significant medical history who was transferred to our institution with increasing shortness of breath, dysphagia, cervical lymphadenopathy and a mediastinal mass identified on chest CT (Figure 1C). A TSH level was below the minimum detection limit of 0.02 uIU/m as well as a free T3 of 12.2 pg/mL (reference range: 3.4-4.8). Biopsies of the anterior mediastinal mass were obtained and showed a type B1 thymoma. Cervical lymph node biopsy revealed dermatopathic lymphadenitis. The patient underwent a robotic thymectomy and a diagnosis of type B1 thymoma was confirmed (Figure 1D). Patient had an uneventful post operative course and was discharged on post operative day 3.

## Discussion

Evaluating an AMM in the setting of GD can be challenging for the clinician. There is no systematic, formal evaluation of how best to manage AMM in the setting of GD [3]. There is substantial variability in the rate of thymic involution as well as thymic size, shape and density even between individuals of the same age [6]. A retrospective study of 109 patients with AMM treated with thymectomy found that all cases with malignancy had either symptoms referable to the thymus or a discrete mass rather than a diffusely enlarged thymus gland [6]. The utility of this information is partially limited by the finding that a “discrete mass” was seen in 32% of thymic hyperplasias and 66% of histologically normal thymus glands [6]. Also, symptoms anatomically referable to enlargement of the thymus (chest discomfort, chest pain and shortness of breath) [6] can frequently also be seen in GD [7]. Prevailing wisdom from several recent case reports of thymic hyperplasia in GD is that caution should be used in evaluating AMM in the setting of GD given the known association with thymic hyperplasia [2-5]. Clinicians are urged to wait until after resolution of hyperthyroidism to follow up AMM in the absence of features concerning for malignancy (invasion, calcifications, cysts or septations) on imaging [2-5]. Reduction of thymic enlargement after achieving a euthryoid state can vary from 2 months up to 2 years [2] which could complicate following up a possibly neoplastic mediastinal mass.

These cases represent two examples of mediastinal neoplasms seen in association with GD. While the first patient represents the first documented case of T-LBL/L seen in association with GD, there are two prior case reports of thymoma occuring with GD [8,9]. On the other hand, occurance of T-LBL/L with other autoimmune diseases is well documented [10-17].

Autoimmunity alters the environment of the immune system with positive selection/expansion of autoreactive T-cells in the thymus. Thymic hyperplasia is a representation of how GD, in particular, alters the microenvironment of the thymus. This is thought to be due to the hyperthyroidism associated with active GD. It is known that thyroid hormones (TH) are able to induce thymic epithelial cell (TEC) proliferation which produces cytokines (including IL-1) [18]. T3 also enhances thymic hormone thymulin secretion by TECs [18]. In turn, cytokines (e.g. IL-1) and thymulin stimulate and enhance thymocyte (immature T-cell precursor) proliferation. Thymocyte differentiation occurs as the cells migrate within thymic lobules interacting with microenvironment [18]. In active GD, thymic microenvironment may be altered due to increased proliferation of TECs driven by TH. It is unclear if thymic environment altered by the state of sustained hyperthyroidism, including increased proliferative drive on maturing thymocytes and, possibly, abnormal differentiation, may contribute to the development of genetic lesions resulting in T LBL/L in susceptible individuals. Likewise it is not unreasonable to postulate that TH driven TEC proliferation may contribute to the pathogenesis of thymoma, which is a neoplasm of thymic epithelial cells. The two patients illustrated not only raise the possibility of association between GD and mediastinal neoplasms but represent a possible reason for caution in using a watch-and-wait approach to anterior mediastinal masses in patients with GD.

## Conclusions

This is the first reported case of T lymphoblastic lymphoma/leukemia and the third reported case of thymoma developing in patients with sustained poorly controlled hyperthyroidism due to Graves disease. Given the known physiologic interactions between thyroid hormones and thymic microenvironment, these cases may indicate a possible link between a sustained hyperthyroidism and the development of thymic neoplasia. In addition, our cases indicate that an anterior mediastinal mass in a patient with active Graves disease may be due to a neoplastic cause, which may require definitive treatment. Caution should be exercised when dismissing a mediastinal mass as benign thymic hyperplasia in patients with active Graves disease.

## Ethical approval

This case report was based on the existing data, and the patients’ identification was kept confidential in this study. This case report does not meet definition of human or animal subject research by University and Medical Center Institutional Review Board of East Carolina University, and no ethical approval was necessary for this study.

## Abbreviations

GD, Graves disease; AMM, Anterior mediastinal masses; T-LBL/L, T-lymphoblastic leukemia\lymphoma.

## Competing interests

The authors declare that they have no competing interests.

## Authors’ contributions

JB performed literature review, patient records review and drafted the manuscript. RJ conceived of the study, and participated in its design and coordination and helped to draft the manuscript. JB and RJ were directly involved in the diagnosis and care of both patients. All authors read and approved the final manuscript.
